# Editorial: Vascular function and aging: a focus on oxidative stress

**DOI:** 10.3389/fphys.2023.1221465

**Published:** 2023-05-30

**Authors:** Gaia Favero

**Affiliations:** ^1^ Anatomy and Physiopathology Division, Department of Clinical and Experimental Sciences, University of Brescia, Brescia, Italy; ^2^ Interdipartimental University Center of Research “Adaption and Regeneration of Tissues and Organs (ARTO)”, University of Brescia, Brescia, Italy

**Keywords:** aging, vessels, oxidative stress, antioxidants, cardiovascular diseases

The collection of studies in the Research Topic “*Vascular function and aging*: *a focus on oxidative stress*” published in Frontiers in Physiology (https://www.frontiersin.org/research-topics/39951/vascular-function-and-aging-a-focus-on-oxidative-stress) would like to report current contributions to the redox state and aging-related alterations at the vascular level.

This Research Topic comprises four original research articles and one study protocol manuscript, providing important contributions to the Research Topic by distinguished experts.

Graton et al. in an *in vitro* study compared antioxidant and/or nicotinamide adenine dinucleotide phosphate oxidase inhibitory properties of two phenolic compounds, apocynin and protocatechuic acid, and their effects on vascular cells (aorta ring) from spontaneously hypertensive rat, a known model of endothelial dysfunction. The Authors concluded that the protocatechuic acid presented greater ability to act as a reactive oxygen species scavenger and seems to have a remarkable and prominent antioxidant capacity. However, apocynin supplementation showed a significant reduction in the vessel contraction and also improved the endothelium-dependent relaxation. This original study so reported that apocynin and protocatechuic acid are potential “tools” for future therapeutic approaches against oxidative stress-related cardiovascular pathological conditions.

Lapi et al. performed an original research study in which they evaluated, in spontaneously hypertensive rats, the effects of physical exercise association with a diet enriched with natural antioxidant phytocomplex (extracted from the apple variety Malus pumila Miller cv. Annurca) in preventing pial microvascular damage correlated with cerebral hypoperfusion and reperfusion injury. In particular, the Authors induced a transitory bilateral common carotid artery occlusion and then evaluated the vascular injury under baseline conditions and after hypoperfusion and reperfusion. Furthermore, in this *in vivo* study, the response to transitory bilateral common carotid artery occlusion in young animals was also analyzed, which are still normotensive, and in aged rats, which presented established hypertension. The Authors concluded that physical exercise and an antioxidant-enriched diet determine protective effects against the hypertension development, even if, to reach better vascular protection, it seems necessary to increase the dosage of diet antioxidants during an intensive physical activity. Unexpectedly, the combination of physical exercise and an antioxidant-enriched diet did not promote greater effects than the single treatment.

Liu et al. presented in an original research study that the oxidative balance score calculated on the basis of 16 lifestyle- and diet-related pro- and antioxidant factors at the final aim to investigate the oxidative balance score and components correlated with endothelial function in Chinese community dwellers. In detail, the factors related to diet were measured by fasting blood samples, and the factors related to lifestyle were evaluated by questionnaires. The Authors concluded that the overall oxidative balance score reflects the body’s oxidative stress status, and it was negatively associated with endothelial function. Furthermore, the dietary-related oxidative balance score was more closely associated with vascular endothelial function with respect to lifestyle-related factors.

Murray et al. performed a study protocol in which a randomized, placebo-controlled, double-blind, parallel group, phase IIa clinical trial which involved healthy older men and women (≥60 years of age) was presented. The clinical trial evaluated the efficacy of the mitochondrial-targeted antioxidant mitoquinol oral supplementation (3 months) in improving vascular function and reducing large elastic artery stiffness (known factors altered in ederly). The Authors reported that this clinical trial is designed to establish the mitoquinol supplementation efficacy and investigate in deep the mechanisms by which this mitochondrial-targeted antioxidant improves age-related vascular injury.

Zhai et al., at the aim to identify new coronary heart disease-related biomarkers, performed a study using untargeted metabolomics to detect differential metabolites in peripheral blood of patients affected by coronary heart disease with various degrees of coronary stenosis. The Authors in this original article presented that 4-phosphopantetheine is associated with the degree of progression of coronary heart disease as well as had inhibitory effects on the formation of atherosclerotic plaque. The Authors reported that this effect might be related to inhibition of reactive oxygen species production and reduction of oxidized-low density lipoprotein uptake by endothelial cells. Together with the aforementioned reported data, the Authors also reported that the synthesis of 4-phosphopantetheine is complicated and expensive, and a less expensive but efficient way to synthesize 4-phosphopantetheine is needed.

The Authors in the present Research Topic would like to emphasize how aging promotes morphological, structural, physiological, and functional changes at the vascular level, even in the healthy status. This Research Topic encourages Researchers to investigate in deep the mechanisms at the basis of many (cardio) vascular- and age-related diseases and identify potential preventive/therapeutic “molecules” which may counteract aging-related vascular injury.

The Research Topic Editor would like to thank all the Authors and the Reviewers who contributed to the success of this Research Topic and the Frontiers in Physiology team for their valuable and constant support.

